# Formulation and Evaluation of Diclofenac Potassium Gel in Sports Injuries with and without Phonophoresis

**DOI:** 10.3390/gels8100612

**Published:** 2022-09-26

**Authors:** Komal Ammar Bukhari, Imran Ahmad Khan, Shahid Ishaq, Muhammad Omer Iqbal, Ali M. Alqahtani, Taha Alqahtani, Farid Menaa

**Affiliations:** 1 Ali-Ul-Murtaza, Department of Rehabilitation Sciences, Muhammad Institute of Medical and Allied Sciences, Multan 60000, Pakistan; 2 Department of Pharmacology and Physiology, MNS University of Agriculture, Multan 60000, Pakistan; 3 Department of Rehabilitation, Bakhtawar Amin Medical and Dental College, Multan 60000, Pakistan; 4 Shandong Provincial Key Laboratory of Glycoscience and Glycoengineering, School of Medicine and Pharmacy, Ocean University of China, Qingdao 266100, China; 5 Royal Institute of Medical Sciences (RIMS), Multan 60000, Pakistan; 6 Department of Pharmacology, College of Pharmacy, King Khalid University, Abha 62529, Saudi Arabia; 7 Departments of Internal Medicine and Nanomedicine, California Innovations Corporation, San Diego, CA 92037, USA

**Keywords:** diclofenac potassium gel, diclofenac sodium gel, phonophoresis, sport injuries, pain

## Abstract

*Background:* Pain remains a global public heath priority. *Phonophoresis*, also known as sonophoresis or ultrasonophoresis, is when an ultrasound is used to maximize the effects of a topical drug. *Purpose:* The objective of this study was to test, in patients injured in sports or accidents (N = 200), the efficacy of diclofenac potassium (DK) 6%, 4%, and 2% formulated gels with and without phonophoresis in comparison with market available standard diclofenac sodium (DS or DN) gel. *Methods:* The patients were enrolled after informed consent. By using the lottery method, 100 patients were randomly segregated into five groups without phonophoresis and repeated similarly with phonophoresis at a frequency of 0.8 MHz, an intensity of about 1.5 W/cm^2^, and at continuous mode (2:1). Group-1 was treated with 6% DK gel, group-2 was treated with 4% DK gel, group-3 was treated with 2% DK gel, group-4 was treated with 4% DS gel and group-5 was given control gel three to four times a week for 4 weeks. The patients were screened by using NPRS and WOMAC scales. They were assessed on the baseline, 4th session, 8th session, 12th session, and 16th session. *Results:* Significant dose-dependently relief was observed in NPRS (Numeric Pain Rating Scale) and the WOMAC (Western Ontario McMaster Osteo-Arthritis) index for pain in disability and stiffness for each group treated with DK gel compared to DS gel. Phonophoresis increased these benefits significantly when used after topical application of DK gel or DS gel, and the dose-dependent effects of DK gel plus phonophoresis were stronger than the dose-dependent effects of DS gel plus phonophoresis. The faster and profounder relief was due to phonophoresis, which allows more penetration of the DK gel into the skin as compared to the direct application of DK gel in acute, uncomplicated soft tissue injury, such as plantar fasciitis, bursitis stress injuries, and tendinitis. In addition, DK gel with phonophoresis was well tolerated. Thus, in this personalized clinical setting, according to the degree of inflammation or injured-induced pain, disability, and stiffness, DK gel 6% with phonophoresis appeared more effective and thus more recommendable than DS gel 6% alone or DS gel 6% combined to phonophoresis.

## 1. Introduction

Sports injuries (e.g., acute ankle sprain, plantar fasciitis, bursitis) are commonly treated with non-steroidal anti-inflammatory drugs (NSAIDs) to minimize discomfort, swelling, and inflammation [1,2]. They have been shown in clinical trials to be effective for the long-term treatment of recurring or chronic illnesses (e.g., back pain, arthritis) as well as for the quick control of severe and acute mild-to-moderate pain and inflammation resulting from injuries such as ankle sprains, musculoskeletal pain, soft tissue, and/or joint injuries [1,2]. Over the counter, topical NSAIDs (1 to 10%, according to the clinical case, and commonly 1 to 2%) are used as an alternative to oral versions [3,4,5]. Topical diclofenac is a typical NSAID that can enter joints, muscles, and synovial fluid through the skin. It spreads and lingers primarily in the target tissues that are inflamed [6,7,8,9]. Clinical trials have also demonstrated that NSAIDs are potent and well-tolerated anxiolytic and anti-inflammatory pain killers for the treatment of both acute and chronic pain conditions, resulting in improved mobility and recovery [3,10,11,12]. The ability of a pertinent NSAID to permeate thoroughly into damaged tissue determines its effectiveness [13]. Efficient permeation and therapeutic response require a delicate balance of lipid and water solubility. The formulation can influence local permeation and pharmacokinetic profile [14,15,16].

**Figure 1:**
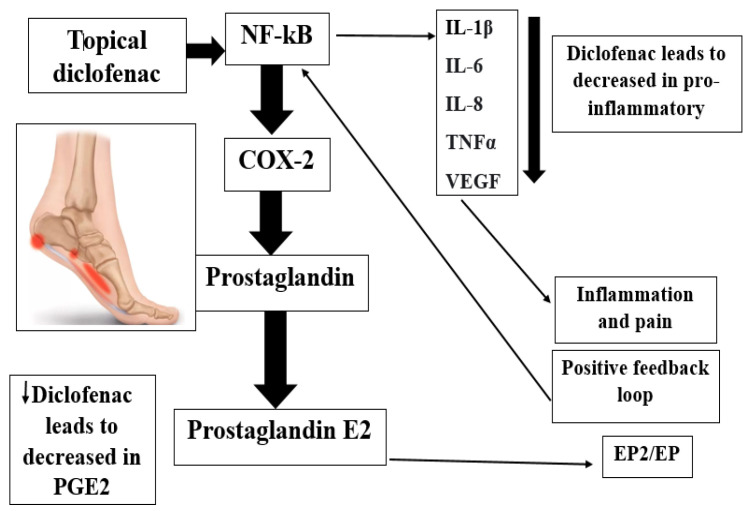
Mechanism of action of diclofenac potassium. **NF-κb:** Nuclear Factor-Kappa B; **COX-2:** Cyclooxygenase-2; **PGE2:** Prostaglandin E2 (dinoprostone); **IL-1β:** Interleukin-1beta (lymphocyte activating factor (LAF)); **IL-6:** Interleukin-6 (B-cell stimulatory factor -2 (BSF-2)); **IL-8:** Interleukin-8 (α chimiokine); **TNF-α:** Tumor Necrosis Factor-alpha; **VEGF:** Vascular Endothelial Growth Factor.

Topical diclofenac, a phenyl acetic acid derivative, which is frequently formulated as sodium salt (DS), has been demonstrated through pharmacokinetic investigations to swiftly enter the skin and reach the underlying tissues (e.g., joints, muscles, synovial) [16,17,18,19,20]. Lecithin, a component that forms micelles, and an aqueous-alcohol micro emulsion serve as the foundation of the 4% (*w*/*w*) diclofenac spray gel formulation (MIKA Pharma GmbH, Speyer, Germany) [16]. Low systemic availability and positive cutaneous penetration were observed [16]. Diclofenac is detected in plasma within 15 min, and due to analgesic and antipyretic properties, efficiency (rapid and targeted actions) and security, diclofenac spray’s is a viable model in severe painful situations [16,17,18]. As per its main molecular mechanism, diclofenac blocks cyclooxygenase-2 (COX-2) [3,10], inhibiting the production of prostaglandins (PGs) from arachidonic acid (AA) (Figure 1). In addition to DS spray gel formulations, research has developed diclofenac patches [17,21,22,23]. Thereby, a semi-occlusive, bioadhesive patch containing 180 mg of diclofenac epolamine (hydroxyethylpyrrolidine), which is comparable to 129.7 mg of diclofenac acid and equivalent to 140 mg of diclofenac sodium, has been prepared [17]. Epolamine (50.3 mg), a pharmacologically inert is used to salify Diclofenac and increase its hydrophilic and lipophilic potencies, making it a viable chemical for topical administration [17]. Unlike DS, DK has only been fabricated for oral administration in the form of a fast-release tablet to deliver better results than oral DS in terms of bioavailability [24,25,26].

A musculoskeletal ultrasound is recommended to diagnose sport injuries-induced heel pain, such plantar fasciitis [27,28,29]. Physiotherapists and orthopedic surgeons often prescribe phonophoresis for the treatment of plantar fasciitis grade I and II [28,29]. Based on the defined test, it incorporates various physiological, physiochemical, and chemical effects, concluding that heat induced by sound waves plays a significant role in the management of different medical conditions; it also produced mechanical results [30,31,32]. Technological advances and high-frequency transducers have made ultrasound technology more desirable than the imaging of abnormal magnetic resonance imaging (MRI) due to its high surface area [30], and recent studies showed that on average, phonophoresis with analgesic gel for the treatment of sports injuries is more effective than local ultrasound alone [32,33,34,35,36]. Although, topical analgesic delivered through phonophoresis in deeper tissues is advised for the treatment of chronic muscular injuries [35,36]and that oral DK displayed a more effective action than oral DS [24,25,26], we failed to find any previous study using DK gel through phonophoresis for the treatment of sports injuries. To the best of our knowledge, DK gel is not available on the market worldwide yet. Herein, we then decided, for the first time, to test the efficacy of various concentrations (2–6%) of DK gels combined or not with phonophoresis in patients suffering from injury-induced pain. The comparison has been carried out with market-available standard DS gel at averaged use concentration (4%).

## 2. Results and Discussion

**Figure 2:**
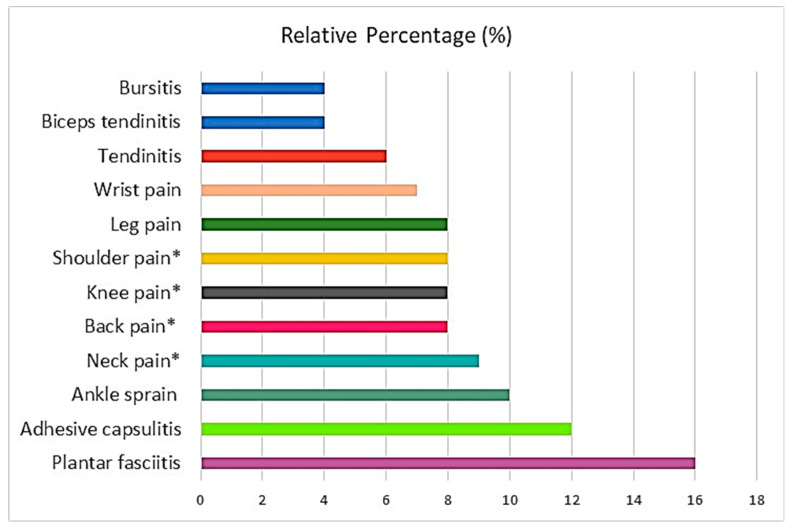
Incidence (%) of patients with sport-related injuries (N = 200) before treatment (which were then subsequently treated). * Means pain in muscles and soft tissues.

In total, 200 patients suffering from sport injuries were enrolled in this study. The patients with planter fasciitis were the most frequent (16%), whereas bursitis (4%) and capsulitis (4%) were the least frequent sport-related injuries (Figure 2). They were followed up on the baseline, at the 4th session, 8th session, 12th session, and at the 16th session of treatment. The quantitative data were presented for mean ± S.D. Statistical insignificance was considered if the *p*-value was less than 0.05. One-way ANOVA was used to determine whether there are any statistically significant differences between the means of two or more independent (unrelated) groups.

**Figure 3:**
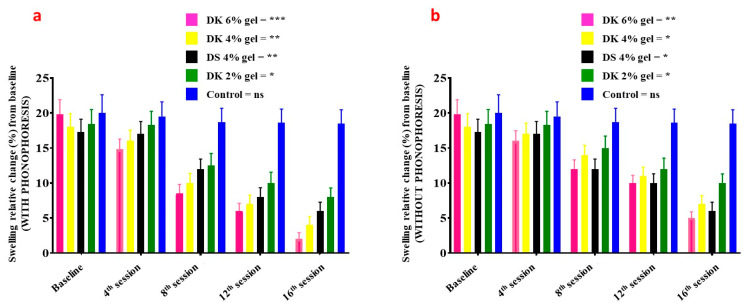
Swelling-related changes on the baseline, 4th session, 8th session, 12th session, and 16th session of Diclofenac potassium (DK) treatment (**a**) with phonophoresis (**b**) without phonophoresis, in sports-injured patients. Diclofenac sodium (DS) was used as a standard control. Control group is the placebo. Statistical significance (*p*-values) is detailed in the main text. ns = no significant; *p* < 0.05 *, *p* < 0.01 **, *p* < 0.001 ***.

Swelling in patients treated with gel combined with phonophoresis was significantly decreasing in a dose-dependent manner (Figure 3a): 6% DK gel (*p* = 0.09–0.02), 4% DK gel (*p* = 0.09–0.03), 2% DK gel (*p* = 0.09–0.05), 4% DS gel (*p* = 0.08–0.02), and for placebo (*p* = 0.09–0.08). Comparatively to patients receiving gel but without phonophoresis, swelling was also significantly decreasing in a dose-dependent manner (Figure 3b): 6% DK gel (*p* = 0.08–0.03), 4% DK gel (*p* = 0.09–0.04), 2% DK gel (*p* = 0.09–0.05), 4% DS gel (*p* = 0.08–0.04), and for placebo (*p* = 0.09–0.08).

**Figure 4:**
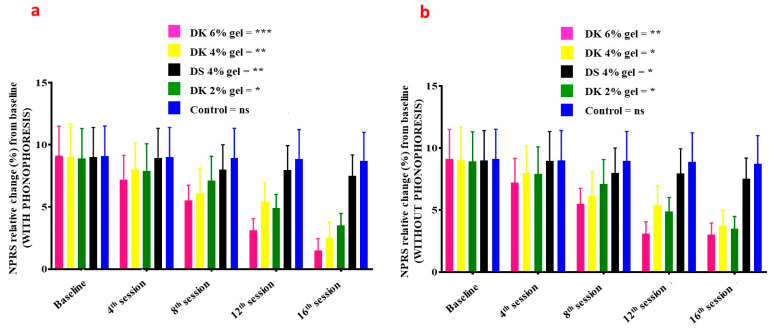
NPRS-related changes on the baseline, 4th session, 8th session, 12th session, and 16th session of Diclofenac potassium (DK) treatment (**a**) with phonophoresis (**b**) without phonophoresis, in sports-injured patients. Diclofenac sodium (DS) was used as a standard control. Control group is the placebo. Statistical significance (*p*-values) is detailed in the main text. ns = no significant; *p* < 0.05 *, *p* < 0.01 **, *p* < 0.001 ***. NPRS: Numerical Pain Rating Scale.

NPRS in patients treated with gel combined with phonophoresis was significantly decreasing in a dose-dependent manner (Figure 4a): 6% DK gel (*p* = 0.009–0.000), 4% DK gel (*p* = 0.08–0.02), 2% DK gel (*p* = 0.08–0.03), 4% DS gel (*p* = 0.08–0.02) and for placebo (*p* = 0.09–0.07). Comparatively to patients receiving gel but without phonophoresis, NPRS was also significantly decreasing in a dose-dependent manner (Figure 4b): 6% DK gel (*p* = 0.009–0.03), 4% DK gel (*p* = 0.09–0.04), 2% DK gel (*p* = 0.08–0.04), 4% DS gel (*p* = 0.08–0.04), and for placebo (*p* = 0.09–0.08).

**Figure 5:**
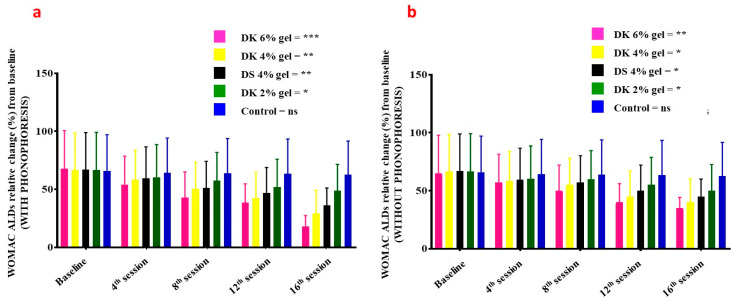
Index of WOMAC ADLs on the baseline, 4th session, 8th session, 12th session, and 16th session of Diclofenac potassium (DK) treatment (**a**) with phonophoresis (**b**) without phonophoresis, in sports-injured patients. Diclofenac sodium (DS) was used as a standard control. Control group is the placebo. Statistical significance (*p*-values) is detailed in the main text. ns = no significant; *p* < 0.05 *, *p* < 0.01 **, *p* < 0.001 ***. WOMAC: Western Ontario and McMaster Universities Arthritis Index, ADLs: activities of daily living.

WOMAC ADLs *(*activities of daily living) in patients treated with gel combined with phonophoresis was significantly decreasing in a dose-dependent manner (Figure 5a): 6% DK gel (*p* = 0.009–0.001), 4% DK gel (*p* = 0.08–0.03), DK gel (*p* = 0.08–0.04), 4% DN gel (*p* = 0.09–0.03), and for placebo (*p* = 0.09–0.07). Comparatively to patients receiving gel but without phonophoresis, WOMAC ADLs was also significantly decreasing in a dose-dependent manner (Figure 5b): 6% DK gel (*p* = 0.009–0.04), 4% DK gel (*p* = 0.08–0.03), 2% DK gel (*p* = 0.09–0.04), 4% DS gel (*p* = 0.08–0.03), and for placebo (*p* = 0.09–0.08).

**Figure 6:**
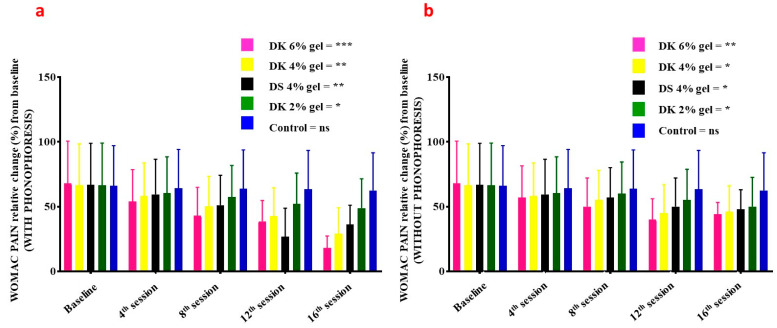
Index of WOMAC pain on the baseline, 4th session, 8th session, 12th session, and 16th session of Diclofenac potassium (DK) treatment (**a**) with phonophoresis (**b**) without phonophoresis, in sports-injured patients. Diclofenac sodium (DS) was used as a standard control. Control group is the placebo. Statistical significance (*p*-values) is detailed in the main text. ns = no significant; *p* < 0.05 *, *p* < 0.01 **, *p* < 0.001 ***. WOMAC: Western Ontario and McMaster Universities Arthritis Index.

WOMAC pain in patients treated with gel combined with phonophoresis was significantly decreasing in a dose-dependent manner (Figure 6a): 6% DK gel (*p* = 0.007–0.001), 4% DK gel (*p* = 0.08–0.02), 2% DK gel (*p* = 0.09–0.05), 4% DS gel (*p* = 0.08–0.03), and for placebo (*p* = 0.09–0.07). Comparatively to patients receiving gel but without phonophoresis, WOMAC pain was also significantly decreasing in a dose-dependent manner (Figure 6b): 6% DK gel (*p* = 0.008–0.04), 4% DK gel (*p* = 0.09–0.04), 2% DK gel (*p* = 0.09–0.05), 4% DN gel (*p* = 0.08–0.04), and for placebo (*p* = 0.09–0.08).

**Figure 7:**
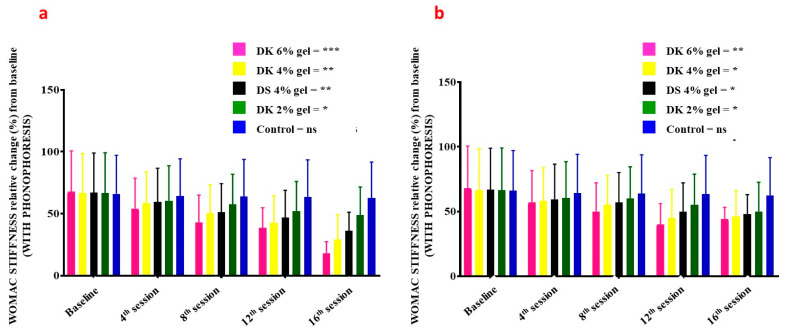
Index of WOMAC stiffness on the baseline, 4th session, 8th session, 12th session, and 16th session of Diclofenac potassium (DK) treatment (**a**) with phonophoresis (**b**) without phonophoresis, in sports-injured patients. Diclofenac sodium (DS) was used as a standard control. Control group is the placebo. Statistical significance (*p*-values) is detailed in the main text. ns = no significant; *p* < 0.05 *, *p* < 0.01 **, *p* < 0.001 ***. WOMAC: Western Ontario and McMaster Universities Arthritis Index.

WOMAC stiffness in patients treated with gel combined with phonophoresis was significantly decreasing in a dose-dependent manner (Figure 7a): 6% DK gel (*p* = 0.008–0.001), 4% DK gel (*p* = 0.09–0.03), 2% DK gel (*p* = 0.08–0.05), 4% DS gel (*p* = 0.08–0.03), and for placebo *p*-value (*p* = 0.09–0.07). Comparatively to patients receiving gel but without phonophoresis, WOMAC stiffness was also significantly decreasing in a dose-dependent manner (Figure 7b): 6% DK gel (*p* = 0.08–0.03), 4% DK gel (*p* = 0.09–0.04), 2% DK gel (*p* = 0.09–0.05), 4% DS gel (*p* = 0.08–0.04), and for placebo (*p* = 0.09–0.08).

Diclofenac is an antipyretic, analgesic, and steroidal anti-inflammatory drug (NSAID) that reduces pain and inflammation by inhibiting the production of prostaglandin (PG) cyclooxygenase-2 [3,10]. In a more detailed way, PGE2 activates the Gq-coupled EP1 receptor, leading to increased activity of the inositol triphosphate/phospholipase C pathway. Activation of this pathway releases intracellular stores of calcium, which directly reduces the action potential threshold and activates protein kinase C (PKC), which contributes to several indirect mechanisms. PGE2 also activates the EP4 receptor, coupled to Gs, which activates the adenylyl cyclase/protein kinase A (AC/PKA) signaling pathway. PKA and PKC both contribute to the potentiation of transient receptor potential action channel subfamily V member 1 (TRPV1) potentiation, which increases sensitivity to heat stimuli. They also activate tetrodotoxin-resistant sodium channels and inhibit inward potassium currents. PKA further contributes to the activation of the P2X3 purine receptor and the sensitization of T-type calcium channels. The activation and sensitization of depolarizing ion channels and inhibition of inward potassium currents serve to reduce the intensity of stimulus necessary to generate action potentials in nociceptive sensory afferents. PGE2 acts via EP3 to increase sensitivity to bradykinin and via EP2 to further increase heat sensitivity. Central sensitization occurs in the dorsal horn of the spinal cord and is mediated by the EP2 receptor, which couples to Gs. Pre-synaptically, this receptor increases the release of pro-nociceptive neurotransmitters such as glutamate, CGRP, and substance P. Post-synaptically, it increases the activity of AMPA and NMDA receptors and produces inhibition of inhibitory glycinergic neurons. Together, these lead to a reduced threshold of activation, allowing low-intensity stimuli to generate pain signals. PGI2 is known to play a role via its Gs-coupled IP receptor, although the magnitude of its contribution varies. It has been proposed to be of greater importance in painful inflammatory conditions such as arthritis. By limiting sensitization, both peripheral and central, via these pathways, NSAIDs can effectively reduce inflammatory pain. PGI2 and PGE2 contribute to acute inflammation via their IP and EP2 receptors. As with β-adrenergic receptors, these are Gs-coupled and mediate vasodilation through the AC/PKA pathway. PGE2 also contributes by increasing leukocyte adhesion to the endothelium and attracting the cells to the site of injury. PGD2 plays a role in the activation of endothelial cell release of cytokines through its DP1 receptor. PGI2 and PGE2 modulate T-helper cell activation and differentiation through IP, EP2, and EP4 receptors, which is believed to be an important activity in the pathology of arthritic conditions. By limiting the production of these PGs at the site of injury, NSAIDs can reduce inflammation.

The goal of this randomized, single-blinded study was to determine in a relatively large cohort population (N = 200 including n = 100 with phonophoresis and n = 100 without phonophoresis) the dose and time (sessions) effects of a newly formulated topical gel product, namely DK gel, with or without phonophoresis, in comparison with DS gel. DS gel, marketed under the name of Voltaren^®^, is known to reduce pain and inflammation at a usage concentration of 1–3%. While 10% DS is generally used for severe clinical cases, it is avoided in patients with certain pathologies such as gastritis or renal/kidneys failure or undergoing vaccination against diseases such as yellow fever or COVID. DK was then used in this study at reasonable concentrations (2–6%). DS represented the standard product, and placebo was used as the control. Phonophoresis is commonly used with a frequency of about 0.8 MHz, intensity 1.5 W/cm^2^, and continuous mode 2:1 [27,37]. Therefore, we applied the same experimental settings in the present study.

To the best of our knowledge and as evoked in the Introduction, this study is the first of its kind. The pertinence of such study resides in the fact that (i) oral DK has shown better analgesic and anti-inflammatory effects compared to DS when they were administered orally; (ii) DS exists in (spray) gels (and patches) for topical application [29] and is commercialized, whereas we failed to find studies related to marketed DK gels; (iii) phonophoresis is commonly used in combination with DS gels for the treatment of musculoskeletal injuries [38,39], and it was proved more effective than DS alone [39,40], but no studies have evaluated phonophoresis in combination with diclofenac derivatives, such as DK.

DK was combined with phonophoresis to increase the pain-relieving effects of the phonophoresis. Indeed, medication particles are pushed deep into the skin tissue by ultrasound waves. A resting membrane potential caused by DK relaxed muscles and phonophoresis increases the penetration of the drug into tissues and circulation, which in turn decreased pain and inflammation [37,38,39,40].

The results showed that (i) DK gel alone is more effective than DS gel alone at equivalent concentration, which is a promising result and offers an alternative option in the treatment of pain; (ii) DK gels 6%, 4%, 2%, or 4% DS gel with phonophoresis (as adjuvant treatment) were more effective than the DK gel 6%, 4%, 2%, or 4% DS gel without phonophoresis, confirming the important role of phonophoresis as adjuvant treatment in pain [35,36]; (iii) the pain reduction (likewise other parameters tested) was dose- and time-dependent, highlighting the necessity of several sessions of cycles of gel therapy with phonophoresis to treat effectively and in a personalized manner patients (according to the degree of suffering from sport injuries, the clinical history of the patient but also from a holistic perspective).

At all time points, the percentage of responders (defined as the percentage of subjects achieving a 50% reduction in swelling of the injured soft tissues for 4 weeks after three to four times treatment in a week) was significantly greater in the group treated with DK gel 6% compared to the other groups. DK gel 6% also resulted in a significantly faster decrease in pain, inflammation, and swelling of the injured soft tissues compared to the other groups. Any comparison of the current study’s results with other intervention programs for soft tissue injuries to assess the effectiveness of DK gel compared to other established products is hampered by differences in study design, inclusion criteria, duration of treatment, and efficacy assessment methods. Furthermore, in recent years, a variety of biomaterials (e.g., patches-based hydrogels, cryogels, nanofibers) together with their distinct physicochemical features have been extensively investigated and developed in the fields of drug-delivery, tissue engineering, medicine, and public health, including disease diagnosis [21,41,42,43,44,45,46,47,48,49]. Thereby, a most recent study in rabbits has reported an injectable thermosenstive hydrogel for dual delivery of diclofenac and avastin (an anti-VEGF) to reduce the inflammation of the corneal neovascularization more effectively [49]. Future studies shall compare DK gels + phonophoresis with hydrogel patches and pain killers-loaded nanomaterials not only in animals but also in a cohort population of patients.

## 3. Conclusions

Group-1 DK gel (6%) with the help of phonophoresis proved highly significant benefits when compared to group-2, group-3, group-4 and group-5 and similar groups without phonophoresis in a good number of patients suffering from sports injuries. Not only did the freshly prepared DK gel 6% quickly help in relieving pain, but it also improved patient mobility because phonophoresis has more penetration of the gel into the skin as compared to direct apply (massage) in acute, uncomplicated soft tissue injuries (e.g., plantar fasciitis, bursitis stress injuries, tendinitis). Strain was also well tolerated. DK gel 6% alone proved more helpful in relieving pain, stiffness, and morbidity than DS gel alone. The promising data open new avenues in the management of musculoskeletal pain due to inflammation or sports injuries because it offers a great alternative to DS in eligible patients. Ongoing clinical studies aim at (i) studying the dose-dependent adverse effects of DK gel at higher concentrations (e.g., 10–15%) in comparison to DS gels, with or without phonophoresis; (ii) developing smart DK hydrogel patches and nanoformulations, and (iii) performing comparisons studies between hydrogel patches, diclofenac nanoformulations and such a present study in different populations.

## 4. Materials and Methods

### 4.1. Study, Patients, and Ethics

A single-blinded, randomized controlled trial (IRCT) was started after approval from the Muhammad Institute of Medical and Allied Sciences’ ethics committee in Multan, Pakistan (2021/IRB/2/PT/01). The study was conducted from November to April 2022. By using the Formula (1), the sample size was determined.

The sample size was 100 subjects calculated with Borkowf formula [50]:

(1) $$\frac{{n=2\sigma^{2}\left(Z1-\alpha +Z1-\beta \right)}^{2}}{{\left(µo-µ\alpha \right)}^{2}}$$

Patients (N = 200) must have met the inclusion requirements to be enrolled in the study. Inclusion criteria considered both genders, adult patients between the ages of 18 and 50, and patients with injuries to the soft tissues (e.g., acute, uncomplicated plantar fasciitis, bursitis, tendinitis, strains, and others mentioned in Figure 2) that occurred between two and eighteen hours before the study enrollment. Exclusion criteria were the following: use of any other medicine, inflammatory or painful disorders as well as fractures and ligament ruptures that were not thought to be amenable to treatment with topical NSAIDS alone, patients in whom NSAIDs treatment may cause serious adverse effects or is not indicated because of other diseases (e.g., kidneys failure, gastritis). The patients were screened by using a numerical pain rating scale (NPRS) and WOMAC index/scale.

**Figure 8:**
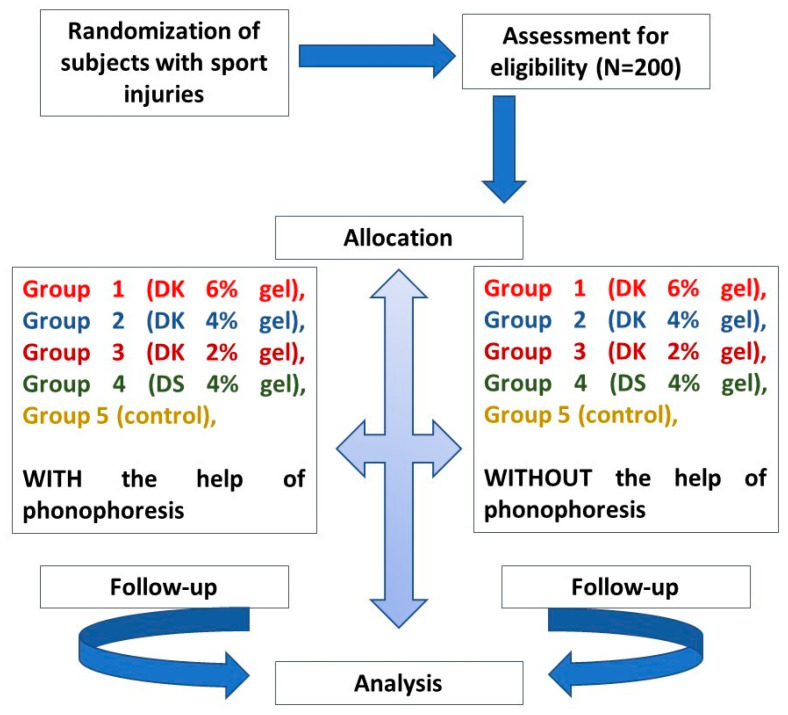
Consolidated Standards of Reporting Trials (CONSORT). DK stands for Diclofenac potassium, and DS means Diclofenac sodium (used as a standard). Control group is the placebo.

**Figure 9:**
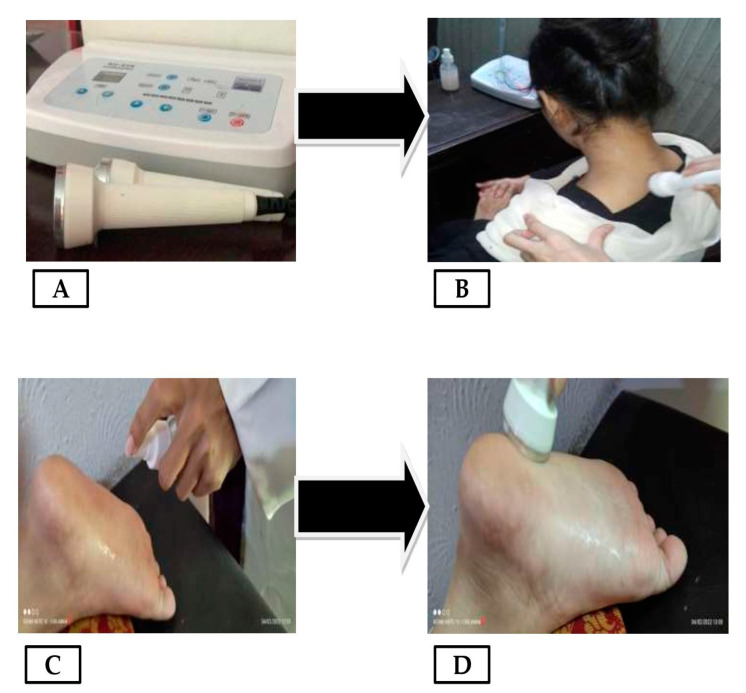
(**A**) Phonophoresis (ultrasound) for the treatment of sport injuries, (**B**) Phonophoresis on a patient with neck pain after applying 4 pumps of DK gel, (**C**) Applying 4 pumps of DK gel on a patient foot, (**D**) Phonophoresis on a patient foot.

All participants signed informed consent. The research was conducted in accordance with the Helsinki Code of Conduct. As shown in Figure 8, out of N = 200 patients, n = 100 were randomly assigned/randomized into each of five groups with and n = 100 without phonophoresis for the treatment of sports injuries. This was completed using the lottery approach. Gel was applied just onto the affected area. Group-1 (n = 20) was given 6% DK gel (1.0–1.4 g containing 40–56 μg, corresponding to daily dose of 96–120 mg DK), Group-2 (n = 20) was given 4% DK gel (0.8–1.0 g containing 32–40 μg, corresponding to daily dose of 96–120 mg DK), Group-3 (n = 20) was given 2% DK gel (0.4–0.8 g containing 16–32 μg, corresponding to daily dose of 48–96 mg DK), Group-4 (n = 20) was given 4% DS gel (0.8–1.0 g containing 50–147 μg, corresponding to daily dose of 50–100 mg diclofenac potassium), and Group-5 (n = 20) was given placebo (water, vehicle only, no active ingredients used here as control group). The treatment was repeated 3–4 times in a week for 4 weeks. Patients were examined on the baseline, day-7, day-14, day-21, and day-28. Phonophoresis (Figure 9A–D), was applied as professionally instructed [51] and set at a frequency of 0.8 MHz, an intensity of about 1.5W/cm^2^, and a continuous mode (2:1) [37]. All the patients were blinded to the treatment.

### 4.2. Statistical Analysis

Data were analyzed by using SPSS version-22 (IBM SPSS, Inc., Chicago, IL, USA). The quantitative data were presented for mean ± S.D. Significance was considered if *p*-value < 0.05. One-way ANOVA was used to determine whether there are any statistically significant differences between the means of two or more independent (unrelated) groups.

### 4.3. Preparation of Diclofenac Potassium Gel

First, 6% DK of analytical grade was purchased from Bukhari’s pharmaceuticals, and the gel was formulated by adding carbazole-940 (6%) into aqueous–methanolic diclofenac potassium (6%). Continuous stirring was used to settle the mixture down until it gained consistency like gel.

### 4.4. Numerical Pain Rating Scale

**Figure 10:**
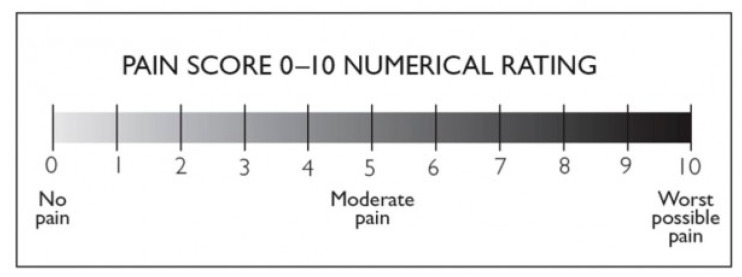
Numerical Pain Rating Scale (NPRS) indicating different intensities of pain: 0 means no pain, 5 reveals moderate pain, and 10 indicates severe pains.

NPRS [52] is often made up of a sequence of numbers and oral anchors that indicate the full range of pain intensity (Figure 10). Patients usually rate their pain on a scale of 0 to 10, 0 to 20, or 0 to 100. They are closely related to other levels of pain and exhibit sensitivity to treatment that is expected to alter pain. Zero stands for “no pain”, while 10, 20, or 100 denotes the extreme of the pain cycle. NPRS can be performed orally or in writing, is basic, easy to grasp, and can be readily controlled and scored. The NPRS’ fundamental flaw is that it lacks the mathematical criteria to be effective. The 11-point numeric scale ranges from 0 to 10, with 0 signifying no pain and 10 reflecting the other extreme degree of pain. We used NPRS in our study before and after the treatment. Then, we checked the scores of pains to improve the stigma of receiving small changes. NPRS numerically modified NPRS was used for scoring pain. The benefits of NPRS include simplicity, reproduction, ease of understanding, and sensitivity to small changes in pain. Five-year-old children who can count and have a certain sense of number (i.e., that 8 is greater than 4) can use this scale. NPRS (whole and each body region) and the specific outcome measure for each region in all 3 levels of change, using the receiver’s operating factor curve. Overall, 64% of NPRS is a valid standard and should be part of a small database of clinical trials, while 14% of NPRS estimates are valid but should only be part of the extended database, 20% of NPRS needs further research to establish credibility and legitimacy before it can be recommended, and 2% of NPRS is not valid or should be used.

### 4.5. WOMAC Index

**Figure 11:**
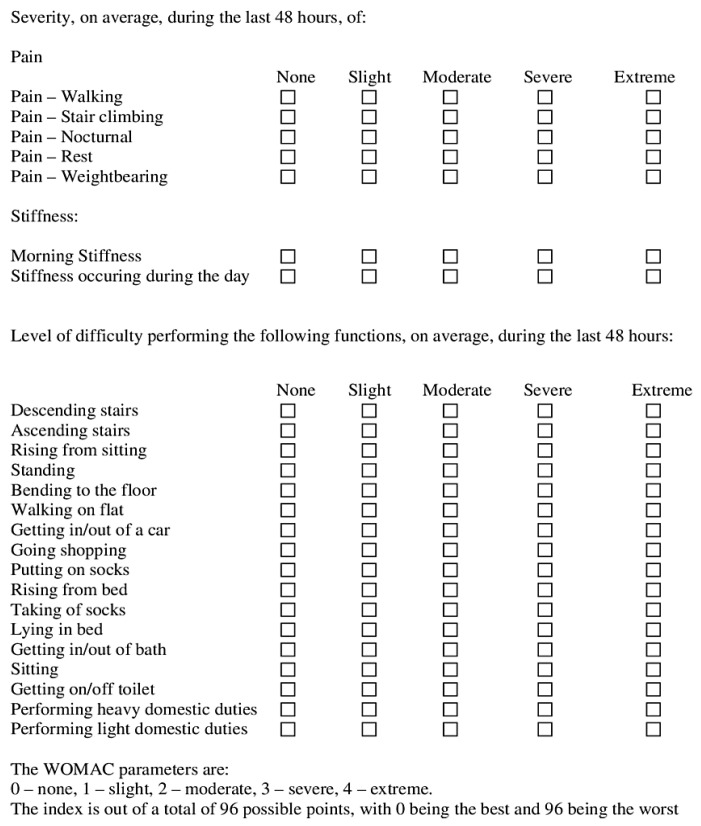
Western Ontario McMaster Osteo-Arthritis (WOMAC) scale/index indicating three parameters: pain, stiffness, and functional activities.

WOMAC [53] is scale estimation for osteoarthritis (OA) of the knee, constituting 24 options in three elements: pain, activities of daily living, and stiffness (Figure 11). However, we assessed only functional disability through this scale. The WOMAC’s test–retest reliability depends greatly on the subscale. Although the pain subscale has not been constant across research, it fulfills the basic criteria. The physical function subscale has higher test–retest reliability and is more reliable. Test–retest reliability for the stiffness subscale is minimal.

## Data Availability

Data may be available upon request to the corresponding author(s).
